# Development of Gluten-Free Cakes Using Protein Concentrate Obtained from Cold-Pressed Terebinth (*Pistacia terebinthus* L.) Oil By-Products

**DOI:** 10.3390/foods14061049

**Published:** 2025-03-19

**Authors:** Muhammed Ozgolet, Salih Karasu, Muhammed Zahid Kasapoglu

**Affiliations:** 1 Department of Food Engineering, Faculty of Chemical and Metallurgical Engineering, Yildiz Technical University, Davutpasa Campus, Esenler 34210, Istanbul, Turkey; skarasu@yildiz.edu.tr; 2 Istanbul Teknokent, Istanbul University-Cerrahpaşa, Teknokent Building, Istanbul 34320, Avcılar, Turkey; muhammed.kasapoglu@iuc.edu.tr

**Keywords:** terebinth seed, protein isolate, gluten-free, oxidative stability, cake, batter rheology, oilcake

## Abstract

The present research aimed to incorporate terebinth seed protein into gluten-free cakes in order to increase their protein content and improve their technological properties. The terebinth protein replaced the rice flour–corn starch mixture used in control cakes at varying levels (3%, 6%, 9%, and 12%). The rheological properties of the cake batters were evaluated, along with the physicochemical attributes, textural properties, sensory attributes, and oxidative stability of the baked cakes. As the protein concentration increased, the consistency index of the cake batters also increased. All batters showed shear-thinning behavior, indicating pseudoplastic fluid behavior, and showed a viscoelastic nature reflected by the storage modulus (G′) exceeding the loss modulus (G″). Both G′ and G″ values increase with increasing protein content. The softest texture was observed in the control cake produced with wheat flour, followed by the cakes with 3% and 6% protein addition, while higher protein levels (9% and 12%) resulted in firmer cakes. Furthermore, oxidative stability improved with a higher level of protein. The addition of protein did not negatively affect sensory quality across all measured parameters. This study demonstrates the potential of terebinth protein to enhance the protein content and oxidative stability of gluten-free cakes that maintain their sensory attributes.

## 1. Introduction

Rising pressures such as resource overexploitation, mismanagement, and climate change necessitate a shift to a circular economy focused on waste reduction and resource optimization. In this model, waste is repurposed into valuable inputs. The oilseed industry generates nutrient-rich by-products that are not fully exploited. Incorporating oilseed meal or press cake into food production contributes to sustainability and advances circular economy principles [1]. This approach not only reduces waste but also provides a valuable source of protein, contributing to the development of affordable, nutrient-rich products [2,3]. Many studies have been conducted to develop new inexpensive protein sources using oilseed-processing by-products [4,5,6]. In addition, proteins extracted from cold-pressed oil by-products can be employed to enhance the functional qualities of food items [7,8]. The demand for cold-pressed oilseed cakes produced via mechanical pressing is currently high due to the simplicity of the equipment required, which can be utilized directly on farms and in rural areas. However, the direct incorporation of oilseed cakes into human or animal nutrition is constrained by the presence of antinutritional factors, which adversely affect organoleptic properties, protein digestibility, and the bioavailability of macro- and microelements [9]. The extraction of protein isolates is an effective method for mitigating antinutritional factors, as it involves the use of high pH conditions that facilitate the removal of these antinutrients [10,11].

An estimated 6% of people worldwide suffer from non-celiac gluten sensitivity (NCGS), and 1% of people worldwide have celiac disease [12]. Additionally, a significant trend has emerged among health-conscious individuals for gluten-free products. This growing request for gluten-free foods has spurred interest in the development of novel gluten-free products [13]. Currently, many gluten-free (GF) products contribute to nutritional inadequacies in the diet, as they often contain high levels of carbohydrates and are deficient in essential nutrients [14].

Terebinth (*Pistacia terebinthus* L.), a member of the *Anacardiaceae* family, is a plant species native to the Mediterranean region, encompassing approximately 20 species. The fruits of terebinth are harvested between August and October and commercially distributed through markets, herbalists, and spice stores [15]. For several thousand years in southern Turkey, terebinth fruits have been utilized as a traditional ingredient in baking, serving as a raw material for the production of specialty village bread and coffee [16]. Studies have demonstrated the bioactive properties of terebinth, including its hypolipidemic effects, potential for atherosclerosis prevention [17], anti-diabetic properties [18], its ability to combat oxidative stress [19], and protective effects against diseases such as cancer [20] and Alzheimer’s disease [21]. Due to its significance, the potential distribution area of terebinth in Turkey has been investigated. Currently, the total area classified as suitable and highly suitable for its distribution is 288.896 km^2^. However, this area is expected to decrease in the future due to climate change [22]. Terebinth seed was differentiated from other oilseeds due to their high oil content (43.3%) [23], significant oleic acid composition (51.2–67.5%) [24], and abundance of phenolic compounds [25]. In a previous study, among five different unconventional seed oil (hemp, radish, terebinth, stinging nettel and laurel), terebinth oil displayed the highest α-tocopherol amount and showed superior oxidative stability with an induction period of 37.55 h [23]. Moreover, pharmaceutical industries widely utilize bioactive compounds derived from plants for applications in medicine, food flavoring, and cosmetic formulations [26]. Natural bioactive compounds are increasingly regarded as viable alternatives to synthetic pharmaceuticals due to their reduced adverse effects [27,28]. Hence, terebinth oil presents itself as a natural alternative to synthetic drugs. Unveiling the health-promoting properties of both terebinth and its seed oil underscores their potential as valuable commercial products and is expected to contribute to the continued expansion of terebinth cultivation [29]. Several studies used terebinth seeds for enrichment in food formulation such as biscuits [30] and cookies [31]. However, terebinth seed oil is anticipated to gain wider utilization. Consequently, the valorization of terebinth oilseed by-products and their components, such as proteins and fibers, warrants attention. Therefore, in this study, protein isolate of terebinth oilseed by-product was incorporated into food formulation.

Cakes are popular baked goods that can be enhanced nutritionally through fortification with protein sources. The incorporation of proteins improved the springiness and softness of cakes, enhancing their textural properties and overall sensory appeal [32]. For instance, Matos and Sanz [33] developed gluten-free cakes using rice flour and various protein sources, finding that soy protein isolate adding significantly improved the viscoelastic properties of the cake batter and positively affected the textural characteristics of the final products. Similarly, Shevkani and Kaur [34] enhanced rice flour cakes with up to 12% protein isolate obtained from cowpea, observing marked improvements in the functional properties of rice flour, with the extent of enhancements being contingent on the type and quantity of protein incorporated. However, there is a lack of research exploring the application of terebinth seed and their oilseed processing by-products in foods. In a previous study, terebinth seed protein exhibited high thermal stability thanks to its high β-sheet configuration, as well as favorable emulsifying and foaming properties [35]. Considering the technological properties of TRP, they have the potential to stabilize the water-oil phase and enhance air bubble retention within the batter during baking, even under high-temperature conditions. Hence, the objective of this study is to evaluate the potential application of incorporating protein isolates derived from terebinth seed by-products (TRP) obtained through cold-pressing into gluten-free cake formulations. Specifically, this study seeks to assess the impact of TRP on the rheological properties of gluten-free cake batters, as well as the textural attributes, sensory qualities, and oxidation stability of the resulting cakes.

## 2. Material and Methods

### 2.1. Material

Terebinth protein was isolated from by-products of cold-pressed terebinth seed oil, containing 18.1% protein, 5.4% fat, 7.7% moisture, and 4.4% ash. These by-products were obtained from cold press oil manufacturer (Tazemiz, Mersin, Turkey). Terebinth seed by-product was prepared by grinding the oil cake residue formed after cold pressing of the seed with NF500 cold press oil machine. The milled material was subsequently sieved to a fine flour, and remaining lipids in the flour were extracted using hexane in our laboratory. Cake ingredients were procured from a local supermarket in Istanbul, Turkey. All of the chemicals used were of analytical grade and were obtained from Sigma-Aldrich (St. Louis, MO, USA).

### 2.2. Methods

#### 2.2.1. Formation of Terebinth Seed Protein Concentrate

Proteins from terebinth seed by-products were extracted using alkali extraction followed by acid precipitation. Optimal extraction conditions (pH 8, 50 °C, 60 min) were determined in a previous study [35]. Briefly, the by-products were suspended in water at a 1:10 (*w*/*w*) solid-to-solvent ratio under these conditions, followed by protein precipitate at pH 4. The resulting protein fraction was obtained through centrifugation at 10,000× *g* for 20 min at 4 °C and subsequently freeze-dried at 50 °C for 72 h (Christ Epsilon 1-4 LSC plus, Martin Christ Gefriertrocknungsanlagen GmbH, Osterode am Harz, Germany). The freeze-dried protein powder was then analyzed for its protein, moisture, ash, and lipid content using standard methods [36]. The moisture content was measured by an oven-drying method, ash content by a muffle furnace method, fat content by Soxhlet extraction, and crude protein content by the Kjeldahl method. A nitrogen-to-protein conversion factor of 6.25 was used in the Kjeldahl method. The terebinth protein isolate (TRP) composition was found to be 66.1% protein, 0.7% fat, 7.6% moisture, and 2.8% ash.

#### 2.2.2. Production of Cake Samples

Cakes were produced based on the technique described by Gökşen and Ekiz [37], with slight modifications. Briefly, wheat flour in control cakes (C1) and rice flour–corn starch mixture in test cakes (C2) were partially substituted with TRP at levels of 3–12%, as outlined in Supplementary Table S1. As a hydrocolloid, xanthan gum was preferred due to its exceptional rheological properties. It was reported that XG enhances CO_2_ retention and contributes to increased specific volume in bakery products [38]. Tubari et al. [39] claimed that XG prevents cake collapse during baking. The concentration of xanthan gum and added TRP was determined based on preliminary trials. The concentration of xanthan gum was optimized to achieve a consistency comparable to the batter with wheat flour. Similarly, protein concentration was adjusted to maintain a flowable consistency in the mixing chamber. Excessive protein addition was observed to hinder the processability of the batters. Initially, eggs were broken and well-mixed using a stand mixer (KitchenAid, Benton Harbor, MI, USA), followed by the gradual addition of sugar. After 2 min of rapid mixing, liquid ingredients were incorporated and mixed at medium speed for an additional 2 min. Dry ingredients were then added, and the mixture was blended at low speed for 2 min. Approximately 50 g portions of cake batter were transferred to a cake pan used for baking with electric oven (FIMAK, Ankara, Turkey) at 180 °C for 30 min.

#### 2.2.3. Rheological Properties of Cake Batters

Cake batters were tested for their rheological qualities using a rheometer that could be manipulated by temperature (MCR302; Anton Paar, Sydney, NSW, Austria) with four distinct tests. All measurements, except for the temperature sweep test, were conducted at a constant temperature of 25 °C. The probe used had a diameter of 25 mm, and the gap between the plates was set to 1 mm. Test conditions were determined by using proposed method with slight modifications [40]. The steady shear test was performed by applying shear rate between 0.1 and 100 s^−1^. The relationship between shear rate and shear stress was fitted to the power-law model (Equation (1)).

(1) $$\tau =K\gamma^{n}$$

In Equation (1), τ represents shear stress (Pa), γ represents shear rate (1/s), K represents consistency index (Pa·s^n^), and n represents flow behavior index. To assess the dynamic rheological characteristic, the linear viscoelastic region was first determined using a stress sweep test. Following this, a frequency sweep test was performed within the linear viscoelastic region over an angular velocity range of 0.1 to 64 rad/s. Variations in the G′ and G″ were evaluated in relation to frequency, with the relationship between frequency and these moduli modeled according to the power-law equations (Equations (2) and (3)).

(2) $$G^{\prime }=K^{\prime }{\left(\omega \right)}^{n^{\prime }}$$

(3) $$G^{\prime \prime }=K^{\prime \prime }{\left(\omega \right)}^{n^{\prime \prime }}$$

In Equations (2) and (3), G′ represents the storage modulus (Pa), G″ represents the loss modulus (Pa), ω represents the angular velocity (rad/s), K′ and K″ represent the consistency indices, and n represents the flow behavior index.

The 3-interval thixotropic time test (3-ITT) was conducted to examine the structural response of cake batters to high shear deformation [41]. This test included three intervals. Initially, a low shear rate of 0.5 s^−1^ was applied for 100 s. In the second interval, a high shear rate of 150 s^−1^ was applied for 40 s to induce batter deformation. In the third interval, the initial low shear condition (0.5 s^−1^) was reinstated, allowing for the assessment of batter structure retention and post-deformation recovery. The extent of deformation from the high shear rate (Equation (4)) and the batter recovery values (Equation (5)) were used for analysis. Additionally, long-term recovery was evaluated by comparing the equilibrium G′ (G_e_) in the third interval with G′ (G_0_) measured immediately after deformation.

(4) $$\;\mathrm{Dr}\;\left(\%\right)=\frac{G_{i}-G_{0}}{G_{i}}\times 100$$

(5) $$\mathrm{Rec}\;\left(\%\right)=\frac{G_{30}}{G_{i}}$$

In Equations (4) and (5), *G*_i_ represents the G′ value in the first interval, *G*_0_ represents the G′ value immediately after exposure to the high deformation, and *G*_30_ represents the G′ value 30 s after the high deformation.

(6) $${\left[\frac{G^{\prime }-G_{e}}{G_{0}-G_{e}}\right]}^{1-n}=(n-1)kt+1$$

In Equation (6), G′ represents the storage modulus (Pa), *k* represents the thixotropic rate constant, *G*_0_ represents an initial storage modulus (Pa) in the third time interval, and *G_e_* is the equilibrium storage modulus.

The temperature dependency of cake batters was examined through a temperature sweep test. In this test, the temperature was gradually increased from 25 °C to 120 °C at a rate of 5 °C per minute, while changes in the storage modulus (G′) were continuously monitored as a function of temperature.

#### 2.2.4. Physicochemical Properties of Cakes

The Kjeldahl method was used for identifying the protein quantity of the cake samples (990.03 AOAC), while the water activity of the samples was recorded at 30 °C using the Novasina Lab Master aw meter. The digital photos were initially cropped, turned to grayscale, and subsequently binarized following the establishment of a threshold. Pore area (mm^2^) and total pore area within the crumb were quantified, allowing for porosity determination as the pore area ratio to total examined area [42]. Cake height was measured with calipers from the base to the highest peak. The volume of the loaf was measured using the method of rapeseed displacement.

The volume-to-weight ratio was used to figure out the specific volume (mL/g). The amount of volume to weight was used to find the specific volume (mL/g). The baking loss was determined through weight measurements of the cake dough before it was baked and then weighing the completed product one hour after it was baked.

#### 2.2.5. Texture Profile Analysis

A texture profile analysis (TPA) was performed on cake crumb using a TA-TX plus Texture Analyzer (Stable Micro Systems, Surrey, UK), following the methodology outlined in a previous study [43]. Cakes were sliced vertically into 20 mm sections. The TPA parameters included a 40% compression, two compression cycles separated by a five-second interval, with pre- and post-test speeds of 1 mm/s and 3.0 mm/s test speed. A cylindrical probe with a 5 g trigger force was utilized. Samples were evaluated at two hours post-baking and after 3 and 7 days of storage. The samples were stored in plastic bags at room temperature until analysis. The measurements were repeated five times.

#### 2.2.6. Oxidation Stability of Cakes

The stability to oxidation of cakes was evaluated using the OXITEST Device (Velp Scientifica, Usmate, MB, Italy) according to a published method [44]. A 20 g sample was placed in the receptacle of the device, and the accelerated oxidation test was conducted at a temperature of 90 °C and a pressure of 6 bar. The redox stability of the sample was quantified by measuring the induction period (IP), reported in hours.

#### 2.2.7. Sensory Analysis

Cakes were evaluated through quantitative descriptive analysis to determine their sensory attributes. The sensory attributes were described to the panelists by a written document (Supplementary Table S2), while sensory descriptors were also explained to the panelists orally. The sensory evaluation was conducted by fifteen trained panelists from the Food Engineering Department at Yildiz Technological University. Descriptive scoring utilized 10 cm unstructured hedonic line scales (Supplementary Figure S1), with the resultant scores being transformed to a 5-point scale through measurement using a ruler.

#### 2.2.8. Statistical Analysis

All findings were reported as mean values along with standard deviations. Unless otherwise specified, all measurements were conducted in triplicate. To identify statistical differences between means, Tukey’s test was applied for multiple comparisons at a significance level of 0.05 using Minitab 18 software (Minitab LLC, State College, PA, USA). The model parameters and R^2^ values were calculated using Statistica software (version 12; Statsoft, Tulsa, OK, USA). The rheological figures were created using Microsoft Excel software (version 2016; Microsoft Office, Redmond, WA, USA).

## 3. Results and Discussion

### 3.1. Rheological Characteristics of Cake Batters

**Figure 1:**
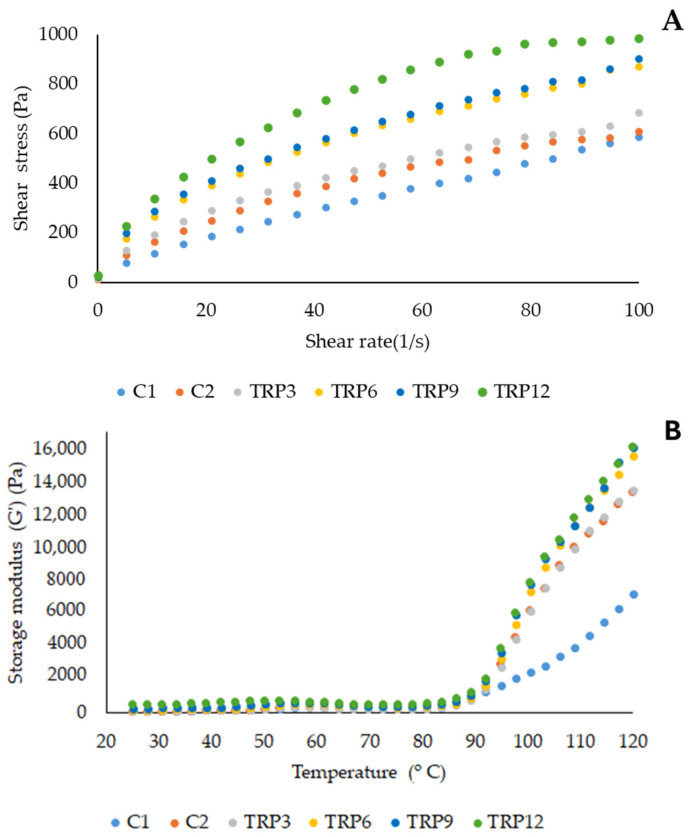
The flow behavior analysis (**A**) and temperature sweep test of cake batters (**B**).

Batter with inappropriate viscosity—either too high or too low—yields cakes with suboptimal quality. High-viscosity batter can restrict gas expansion, resulting in low-volume baked products with dense, crumbly, and less elastic crumb structures. Conversely, low-viscosity batter may cause the over-expansion of air and gas cells, leading to large, collapsed cells and uneven texture, compromising the product’s structural integrity and springiness. The ideal cake batter must possess adequate density to inhibit the escape of air bubbles during baking [4]. The consistency of the cake batter was examined using steady-shear testing. Figure 1 illustrates the relationship between shear stress and varying shear rate. Shear stress values increased with the rising TRP content. The graph’s slope indicates that viscosity dropped as the shear rate rose, demonstrating the shear-thinning characteristics of these cake batters. This property in gluten-free cake batter helps prevent excessive viscosity increases, which could otherwise cause difficulties in batter handling, equipment cleaning, mold filling, and energy consumption during pumping. The power-law model was used to determine the consistency index (K) and flow behavior index (n) of cake batters, as shown in Table 1. TRP-enriched cake batters exhibited K values ranging from 57.10 Pa·s^n^ to 128.78 Pa·s^n^, while the wheat control batter (C1) and gluten-free control batter (C2) had K values of 18.84 Pa·s^n^ and 45.57 Pa·s^n^, respectively. The addition of TRP led to higher K values, attributed to the interaction between cake ingredients. The flow behavior indices (n) of TRP-enriched batters ranged from 0.50 to 0.53, which were lower than that of the C1 control batter. Increasing protein content in TRP cakes did not significantly impact the n values (*p* > 0.05). With n values below 1, these batters display non-Newtonian shear-thinning behavior. TRP-containing cake batters showed significantly lower n values and higher K values compared to control batters, suggesting enhanced pseudoplasticity and higher consistency [45]. The elevated K values in TRP-enriched batters compared to the control samples batter (C1) may be due to the presence of xanthan gum in gluten-free formulations. A similar effect has been observed when combining soy protein and xanthan gum, resulting in elevated viscosity values in gluten-free cake batters, which aligns with our findings [46]. Similarly, Ronda and Oliete [47] reported the n value of corn starch based gluten-free cake batter is lower than wheat starch based cake batter while K value of corn starch based batter is higher than wheat starch based batter. The incorporation of soy protein isolate increased batter consistency in the cake batter. Soy protein enrichment of 10% led to a 2.4-fold increase in G′ value while 20% enrichment caused a 4.1-fold increase in G′. Also, Sahagún and Bravo-Núñez [48] added various protein sources to cake batters and the consistency of all batters increased with the increase in protein source. The addition of 30% pea protein addition increased the viscosity of cake batter from 2.3 to 5.5 Pa.s. This increase was attributed to the water-holding capacity and foaming capacity of the proteins.

**Figure 2:**
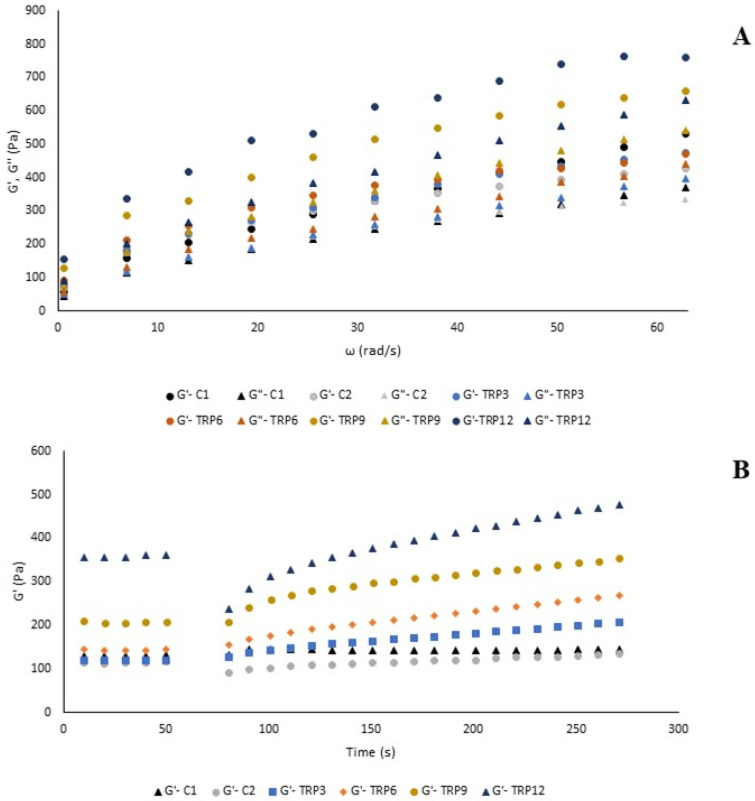
Viscoelastic properties (**A**) and thixotropic properties (**B**) of the cake batters.

Figure 2A shows the changes in the storage (G′) and loss (G″) moduli of the batters with varying frequency. In all batters, the G′ values were higher than the G″ values, indicating a soft gel characteristic and a solid-like structure. As the addition of TRP protein increased, both G′ and G″ values also increased, demonstrating that the addition of TRP further reinforced the viscoelastic structure. These observations aligned with the consistencies in steady-shear test. The G′ reflects the elastic behavior of the batter, while G″ the indicates its viscous properties. A higher storage modulus (G′) indicates a firmer batter that can retain air bubbles and maintain structure during mixing and baking. The loss modulus (G″) reflects the batter’s fluid-like behavior, with a higher G″ signifying more energy loss as heat, affecting flowability and mixing ease. The batter’s viscosity should be optimized to effectively trap gas bubbles during mixing and retain them throughout baking. Previous studies have indicated that both excessively low and high batter viscosities lead to muffins with reduced volume [49]. The power-law model parameters are detailed in Table 1. The K′ values of TRP-enriched cake batters ranged from 76.95 to 163.21 Pa·s^n^, while K″ values ranged from 39.16 to 71.11 Pa·s^n^. The observation that K′ surpassed K″ in all samples indicates a dominating viscoelastic solid nature in the batters. The incorporation of soy and pea proteins into the cake mix enhanced viscoelasticity, yielding stiffer textures, as indicated by increased G′ and G″ values [50].

**Figure 3:**
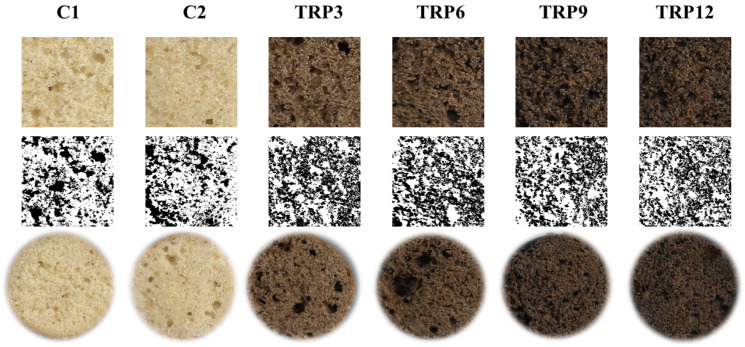
The pore structure of the cake samples.

In a study by Kaur and Singh [51], the addition of various oilseed cakes to cake batters increased viscoelasticity by enhancing G′ and G″, which was connected with a higher protein content and greater water retention capacity of the flours. The G′ values remained consistently higher across all angular velocities, indicating the batters’ ability to retain air bubbles. Additionally, incorporating varying protein isolates into non-gluten cake mixtures further increased viscoelasticity by elevating G′ and G″ values. Similarly, the enhanced viscoelasticity of protein-enriched batters was linked to the proteins’ capacity to absorb free water, facilitating particle movement within the batter matrix [49]. In addition, the combination of protein sources with xanthan gum may lead to increase in both moduli. Soy protein and xanthan gum addition caused an increase in storage modulus at 1 Hz from 115.1 to 982.1 Pa and in loss modulus from 80.5 to 458.04 Pa. The increases in G′ and G″ were attributed to the water-binding capacity of xanthan gum, which decreases the availability of free water. The effect of soy protein isolate was explained by the globulin units of the protein, which function as gel stabilizer. Additionally, the interaction of starch granules with proteins through electrostatic forces, hydrophobic interactions, and hydrogen bonding facilitated the formation of a more stable three-dimensional protein gel network, thereby increasing consistency [52]. It should be noted that the mechanical spectra were performed under LVR conditions, which provide information about the sample’s structure in an unaltered state. Although these tests do not simulate the actual mixing process, they can be correlated with the sample’s behavior when subjected to greater deformations [53]. The increase in solid character with TRP addition did not influence specific volume and height of cakes. A wide range of cake batter consistencies have the ability to form gasses inside cake batters. In addition, although TRP addition affected the flowability of cake batter, the cake batter flowed without the need for any external force when the mixture chamber was inverted. In industrial production, higher pumping energy may be required to transfer TRP-enriched cake batters. The 3-ITT test is a specialized rheological approach to measuring thixotropic characters, offering an effective means of evaluating the structural breakdown and regeneration in food materials. Figure 3 illustrates that all cake batters exhibited thixotropic behavior during the third interval, indicating their ability to regain viscoelastic properties after being subjected to high shear-rate deformation. This finding suggests that all batter samples can preserve their viscoelasticity under intense deformation under shear force. The model parameters for 3-ITT are displayed in Table 2. All batters exhibited Ge/Go values greater than 1, signifying their capacity to recover structure following high-shear deformation. As anticipated, in the first interval, the addition of TRP increased the G′ values of the cake batters. In the second interval, high shear deformation was applied to cake batters to examine their deformation and recovery. In the third interval, the G′ values of C2 and TRP12 decreased, with %Def values of 22.8% and 34.3%, respectively, indicating a loss of batter integrity. The %Rec values revealed that C2 and TRP12 were unable to fully recover their structures within 30 s of high shear, as their %Rec values were below 100%. In contrast, C1, TRP3, TRP6, and TRP9 showed no structural degradation during the second interval. Similarly, 12% of milk thistle seed protein enrichment caused a decrease in %Rec of gluten-free cake batter from 144.8% (3% addition) to 91.9% among protein-added batters in a previous study [7]. In another study, higher ratios of protein addition to cake formulations resulted in significant deformation compared to formulations without added protein [4]. Furthermore, all TRP-enriched and C2 batters demonstrated higher Ge/Go values than C1, indicating a greater increase in G′ during the third interval. This demonstrated that the long-term resting in the third interval after high shear deformation allowed all gluten-free cakes to recover their structures. This may be due to xanthan gum in gluten-free cake batters. In a previous study, wheat-sorghum composite dough with xanthan gum was evaluated for its elastic behavior using a high-strain relaxation test [54]. The findings revealed that xanthan gum addition to composite dough increased dough elasticity, which is attributed to the gelling and stabilizing effect of xanthan gum.

Figure 1B illustrates the variations in storage modulus (G′) as the temperature rises from 25 to 120 °C. The temperature-dependent flow behavior of cake batters provides insight into their pasting characteristics, which are largely influenced by the composition of the flour [34]. The temperature sweep test is relevant for evaluating the properties of the resulting muffins because it simulates the thermal transitions that occur during baking. It provides insights into starch gelatinization, protein network formation, and overall structural changes, which are key factors influencing the texture of the final product. Upon heating, G′ rises when starch granules interact with water and enlarge due to gelatinization but subsequently declines when the granules disintegrate at elevated temperatures. When the temperature surpassed 80 °C, both the storage and loss moduli rose in all cake batters, corresponding to starch gelatinization. In this study, G′ values initially increased with the temperature, consistent with findings from Singh and Kaur [55], where the rise in G′ at lower temperatures was linked with interaction of proteins [14,46]. Subsequently, the G′ values started to drop until the starch reached its gelatinization temperature. This reduction in G′ is likely due to protein denaturation or dissociation, as well as the breakdown of the starch’s three-dimensional structure [32]. At temperatures higher than 90 °C, the G′ of C1 batter differentiated from the others. In a previous study, Mukprasirt and Herald [56] reported that rice and corn flours showed higher viscosity compared to wheat flour at high temperatures due to a high level of starch content. The stiffness of gluten-free cakes may be related to high G′ at the temperatures higher than 90 °C. In addition, during storage, gluten-free cakes displayed faster increase in hardness. This may be attributed to the recrystallization of these gelatinized starch molecules. Salazar et al. [57] reported that the retrogradation of starch, characterized by the recrystallization of gelatinized starch during cooling and storage, may ultimately contribute to texture hardening and moisture loss.

### 3.2. Cake Samples’ Physicochemical Properties

The physicochemical parameters of the cakes are displayed in Table 3. Although the cakes with TRP exhibited increased height, no significant differences were observed in their specific volume compared to the control cakes. Moreover, no significant difference was observed between the control cakes, C1 and C2. This is likely attributed to comparable porosity in the cake crumb. Both cake batters had the lower consistency indices compared to TRP-added cake batters. Therefore, the produced gasses may not have been retained in cake batters. The incorporation of TRP led to the formation of cakes with greater porosity than the control group. Likewise, Shevkani and Kaur [34] reported that proteins with strong emulsifying and foaming properties positively impacted cake quality by reducing interfacial tension, which enhanced batter aeration and crumb porosity. From Figure 3, the large collapsed bubbles were observed in TRP3 and TRP6 cake crumbs, probably leading to the highest porosity values. Large bubbles can be related to distinctive gas holding ability of TRP3 and TRP6 due to their moderate consistency compared to other batter (C1, C2, TRP9, and TRP12). At the initial stage of baking, TRP3 and TRP6 batter consistency can be adequate to retain gasses inside cake batter. However, increasing temperature caused protein denaturation (Figure 1B) and decreased the integrity of these two batters, leading to large bubble collapse. Higher levels of protein addition (TRP9 and TRP12) adversely affected the circularity of the pores. The control cakes exhibited smaller pore perimeters, indicating that they contained more but smaller pores. The higher consistency of TRP-added cakes probably led to retention of larger and different size of air bubbles, resulting in larger and non-circular pore structure. C1 and TRP-enriched cakes exhibited higher protein content than C2. This can be attributed to the incorporation of TRP in gluten-free cakes, as well as the higher protein content of wheat flour compared to the rice flour–starch mixture used in the gluten-free cake formulation. TRP9 and TRP12 cakes had greater protein content than the C1 cake with wheat flour. No significant differences were observed in the water activity values across all cake samples. Water activities of cake samples decreased from 0.78–0.79 to 0.74–0.75 after seven days storage. There was no visible mold growth on the surface of the cakes. Similarly, in a previous study, the water activity of muffins produced without any preservatives declined from 0.77 to 0.76 in 8 days and to 0.74 in 16 days. There was also no visible mold growth in this period [58]. However, beyond 18 days, visible mold growth was observed. Therefore, natural or synthetic preservatives can be added to the cake formulations to extend shelf-life.

### 3.3. Textural Properties of Cake Crumbs

Textural characteristics of the cakes are presented in Table 4. The hardness of the C1 cake was lower than other cakes during storage. Also, the addition of 9% and 12% TRP caused the highest hardness values on the first day. Similarly, the addition of whey protein to the cake formulation caused an increase in hardness which is attributed to their denaturation and subsequent aggregation due to high temperature [59]. During storage, all TRP-enriched batters except TRP3 exhibited similar trend in hardness values with C2 cake. TRP3 cake displayed softer crumb structure than other gluten-free cakes. It was reported that 3% of soy protein isolate addition declined the increase rate of hardness compared to gluten-free control cake during storage [60]. In another study, a higher level of chickpea protein addition caused stiffer crumb structure and the increase in hardness was attributed to the formation of cross-linked molecules between xanthan gum and proteins [61]. The springiness values of cake crumbs were indifferent on all analyzed days (*p* > 0.05). Likewise, Dhillon and Kour [62] utilized oyster mushroom powder (OMP) as a novel protein source in rice-based gluten-free cake formulation. They reported that OMP addition increased hardness while there was no significant change in springiness values. The difference between the cohesiveness values of cake crumbs was insignificant on the first day; however, gluten-free cakes lost their integrity during storage, leading to lower cohesiveness values. Researchers in [63] claimed that glutenin reduced retrogradation probably due to interaction with amylose during starch gelatinization and recrystallization. In starch-containing batters, amylose transforms from an amorphous to a more ordered and crystalline state in an optimal starch gel. Therefore, the observed decrease in cohesiveness may potentially be attributable to the proteins inhibiting amylose’s ability to reorder and declined retrogradation [64]. Chewiness is defined as the amount of energy required to masticate a product until it reaches a swallowable consistency. The chewiness value is calculated by multiplying the values of cohesiveness, springiness, and hardness. The chewiness values of TRP9 and TRP12 were the highest on the first day due to the increased hardness of cake crumbs. However, there was no significant difference in chewiness after 7 days of storage among all cake crumbs. This is associated with the firmer and more crumbly structure (low cohesiveness) of gluten-free cakes due to the aforementioned reasons. The formation of xanthan gum–protein cross-links, along with an increased rate of starch retrogradation due to the more ordered structure of amylose in the starch gel within gluten-free cake crumbs, accounts for the similarity in chewiness values observed on the 7th day of storage. The resilience of the cakes decreased during storage; however, the resilience values of all cakes did not differentiate from each other on all analyzed days (*p* > 0.05).

### 3.4. Oxidative Stability of the Cake Samples

The induction periods (IP) for control and TRP-added cakes at 90 °C and 6 bar are presented in Table 3. The IP of TRP-added cakes (ranging between 10:30 and 13:24 h) was lower than that of the control 1 and 2 cakes (C1: 10:60 h, C2: 10:31), indicating that TRP increased the oxidative stability of the cakes. It was reported that emulsifiers with high molecular weight can act as a protective barrier, reducing interactions between lipids and aqueous-phase prooxidants, thereby slowing the rate of lipid oxidation [65]. TRP proteins have a molecular weight of 20–36 kDA and can act as emulsifiers thanks to their amphiphilic nature [35]. In the same study, TRP was considered due to its high solubility around neutral pH. This may facilitate the formation of a protective membrane that encapsulates lipids, thus inhibiting the oxidation of lipid droplets. Faraji et al. [66] found that soy protein isolates (SPI) exhibited a greater protective effect against oxidative degradation in emulsions compared to milk-derived proteins, such as whey proteins and caseins attributed to sulfhydryl groups and amino acids that can scavenge free radicals. A previous study revealed that the antioxidant property of albumin proteins was associated with synergistic effect of this proteins with phenolic compounds [67]. This study revealed that TRP has the potential to increase the shelf-life of cake samples due to probable declining lipid peroxidation during storage. It was claimed that toxic carbonyl lipid peroxidation products form in cake production [68]. Further studies could focus on the effect of TRP on the formation of toxic compounds in cakes resulting from lipid peroxidation. The antioxidant potential of TRP proteins have been demonstrated in a previous study [35] and TRP offered comparable antioxidant activity to other plant-based proteins. It should be noted that TRP also consists of a non-protein portion, hence, the antioxidative effect of TRP may be related to the compounds that have antioxidant properties or their synergistic impact with proteins. Although this study showed the protective effect of TRP enrichment on the lipid oxidation of the cakes, a more detailed study to reveal antioxidant activity of TRP ought to be conducted. 

### 3.5. Sensory Analysis

The sensory properties of the cakes were assessed based on their textural attributes. These characteristics are presented in Table 5. The addition of TRP showed a comparable likeness with C1 and C2 cakes. There is no significant difference between TRP-added cakes and control cakes (*p* > 0.05). Although the color of the cakes darkened with TRP addition due to natural pigment and alkalization process, this increase did not disturb panelists. The common consumption of cakes containing cocoa powder may be reason for this likeness. When it comes to overall quality, despite the fact that cakes with TRP added scored better than those with C2, there was no discernible difference (*p* > 0.05). In a previous study, the addition of chickpea protein until 10% did not unfavorably influence cake sensory characteristics [69]. Moreover, Banu and Patrașcu [70] added protein-based emulsions (6% protein) including soy, lupin, and yeast proteins into cakes and they reported that there was no significant change in sensory attributes such as taste, flavor, and overall acceptability, consistent with our results. The sensory analysis indicated that the fortification of cakes with up to 12% TRP does not negatively impact the sensory attributes of the cakes.

## 4. Conclusions

This study investigated the influence of terebinth seed proteins on cake batter and quality. The addition of protein enhanced the viscoelastic properties of cake batters, which all exhibited shear-thinning characteristics and resembled a solid-like gel structure. The TRP9 and TRP12 batters with the most consistent rheological properties in both steady and dynamic tests produced cakes with the highest hardness on the first day. This finding was supported by the temperature sweep test, which revealed high G′ values at elevated temperatures, indicating enhanced structural development during baking. All cake batters exhibited thixotropic behavior with high recovery rates, indicating an improved ability of the batter to regain its viscoelastic properties, which is beneficial for batter handling and deposition. The incorporation of TRP in cakes resulted in a more porous structure while simultaneously enhancing their oxidative stability. Sensory analysis demonstrated that TRP proteins can produce cake samples with acceptable taste, flavor, and overall quality comparable to the control cakes. Consequently, TRP proteins can be utilized to increase the amount of protein and oxidative stability of cakes without compensating textural and sensory characteristics. The textural properties of cakes produced with wheat flour can be achieved by using the interaction of TRP protein with different gums or TRP protein hydrolysates in further studies. Additionally, further research should explore how consumers perceive the use of industrial by-products for protein enrichment in cake production. This study offers a method for the enrichment of gluten-free cakes with a distinctive ingredient, directly addressing the consumer need for high protein-containing products in the GF market.

## Figures and Tables

**Table 1:** Power-law model parameters indicating flow behavior and viscoelastic properties of cake batters.

	K	n	R^2^	K′	n′	R^2^	K″	n″	R^2^
C1	18.84 ± 1.80 ^e^	0.74 ± 0.04 ^a^	0.997	44.84 ± 1.00 ^e^	0.59 ± 0.02 ^a^	0.995	40.97 ± 1.61 ^d^	0.55 ± 0.03 ^a^	0.998
C2	45.57 ± 3.31 ^d^	0.53 ± 0.03 ^b^	0.999	80.92 ± 2.53 ^d^	0.36 ± 0.02 ^c^	0.997	46.71 ± 0.85 ^c^	0.49 ± 0.03 ^a^	0.998
TRP3	57.10 ± 1.87 ^c^	0.53 ± 0.02 ^b^	0.999	76.95 ± 1.93 ^d^	0.43 ± 0.02 ^b^	0.998	39.16 ± 2.30 ^d^	0.54 ± 0.02 ^a^	0.996
TRP6	80.76 ± 4.77 ^b^	0.52 ± 0.03 ^b^	0.999	107.97 ± 2.53 ^c^	0.37 ± 0.01 ^c^	0.998	42.68 ± 1.20 ^cd^	0.55 ± 0.02 ^a^	0.994
TRP9	90.05 ± 2.84 ^b^	0.50 ± 0.02 ^b^	0.999	128.25 ± 6.88 ^b^	0.39 ± 0.03 ^bc^	0.997	63.74 ± 3.16 ^b^	0.51 ± 0.03 ^a^	0.997
TRP12	128.78 ± 6.95 ^a^	0.50 ± 0.04 ^b^	0.991	163.21 ± 8.01 ^a^	0.38 ± 0.02 ^bc^	0.997	71.11 ± 2.45 ^a^	0.52 ± 0.04 ^a^	0.997

K, K′, and K″ represent the consistency indices and n, n′, and n″ represents flow behavior indices. Data are presented as means ± SD of replicates. Distinct lowercase letters in the same column denote statistical significance (*p* < 0.05).

**Table 2:** The model parameters for 3-ITT.

Sample	G_0_	G_e_	G_e_/G_0_	*k* × 1000	R^2^	%Def	%Rec
C1	125.62 ± 2.03 ^d^	148.20 ± 6.60 ^d^	1.18 ± 0.04 ^e^	58.6 ± 1.0 ^a^	0.961	-	110.7
C2	89.50 ± 3.00 ^e^	182.48 ± 7.73 ^c^	2.04 ± 0.02 ^d^	4.4 ± 0.3 ^d^	0.988	22.8	92.2
TRP3	126.35 ± 3.19 ^d^	420.06 ± 5.37 ^b^	3.32 ± 0.04 ^b^	1.8 ± 0.3 ^e^	0.997	-	124.4
TRP6	153.27 ± 4.78 ^c^	616.92 ± 7.71 ^a^	4.03 ± 0.07 ^a^	1.6 ± 0.2 ^e^	0.999	-	126.2
TRP9	196.41 ± 3.12 ^b^	413.15 ± 6.43 ^b^	2.10 ± 0.02 ^d^	11.8 ± 0.5 ^b^	0.991	-	129.2
TRP12	223.53 ± 7.55 ^a^	622.87 ± 7.51 ^a^	2.79 ± 0.07 ^c^	7.9 ± 0.7 ^c^	0.995	34.3	90.6

*G*_0_ represents an initial storage modulus (Pa) in the third time interval, *G_e_* is the equilibrium storage modulus, *k* represents the thixotropic rate constant. Data are presented as means ± SD of replicates. Distinct lowercase letters in the same column denote statistical significance (*p* < 0.05).

**Table 3:** Physicochemical properties and oxidative stability of cake samples.

	Specific Volume (cm^3^)	Height (cm)	Bake Loss (%)	Porosity (%)	Circularity	Perimeter	Protein (%)	1st Day	A_w_ 4th Day	7th Day	IP (h)
C1	2.51 ± 0.03 ^a^	45.5 ± 0.8 ^bc^	11.5 ± 0.5 ^a^	38.03 ± 1.51 ^c^	0.849 ± 0.004 ^b^	18.23 ± 0.23 ^d^	5.43 ± 0.03 ^c^	0.78 ± 0.01 ^a^	0.76 ± 0.02 ^a^	0.74 ± 0.02 ^a^	10.60 ± 0.44 ^c^
C2	2.51 ± 0.04 ^a^	44.7 ± 0.2 ^c^	12.2 ± 0.6 ^a^	37.22 ± 1.79 ^c^	0.856 ± 0.003 ^ab^	16.89 ± 0.85 ^d^	3.67 ± 0.05 ^e^	0.79 ± 0.01 ^a^	0.76 ± 0.02 ^a^	0.75 ± 0.03 ^a^	10.31 ± 0.16 ^c^
TRP3	2.54 ± 0.05 ^a^	46.9 ± 0.5 ^a^	12.3 ± 0.2 ^a^	64.41 ± 2.02 ^a^	0.871 ± 0.007 ^a^	43.60 ± 2.16 ^ab^	5.16 ± 0.07 ^d^	0.78 ± 0.02 ^a^	0.75 ± 0.01 ^a^	0.75 ± 0.02 ^a^	10.30 ± 0.23 ^c^
TRP6	2.57 ± 0.04 ^a^	47.7 ± 0.8 ^a^	12.3 ± 0.1 ^a^	64.47 ± 3.23 ^a^	0.801 ± 0.008 ^c^	44.71 ± 3.11 ^a^	5.55 ± 0.07 ^c^	0.78 ± 0.01 ^a^	0.75 ± 0.02 ^a^	0.74 ± 0.02 ^a^	12.27 ± 0.27 ^b^
TRP9	2.58 ± 0.06 ^a^	47.9 ± 0.5 ^a^	12.1 ± 0.6 ^a^	52.49 ± 2.34 ^b^	0.799 ± 0.007 ^c^	39.07 ± 1.40 ^bc^	6.07 ± 0.05 ^b^	0.78 ± 0.01 ^a^	0.75 ± 0.03 ^a^	0.74 ± 0.02 ^a^	12.45 ± 0.11 ^b^
TRP12	2.60 ± 0.05 ^a^	48.4 ± 0.9 ^a^	12.3 ± 0.3 ^a^	50.93 ± 1.70 ^b^	0.785 ± 0.008 ^c^	37.21 ± 1.46 ^c^	6.38 ± 0.07 ^a^	0.78 ± 0.02 ^a^	0.74 ± 0.01 ^a^	0.74 ± 0.03 ^a^	13.24 ± 0.24 ^a^

Data are presented as means ± SD of replicates. Distinct lowercase letters in the same column denote statistical significance (*p* < 0.05).

**Table 4:** Texture profile analysis of cakes fortified with terebinth seed protein isolate.

Parameters	Storage Day	C1	C2	TRP3	TRP6	TRP9	TRP12
Hardness (N)	1	7.03 ± 0.09 ^c^	8.30 ± 0.21 ^b^	7.93 ± 0.11 ^b^	8.14 ± 0.28 ^b^	11.76 ± 0.22 ^a^	11.46 ± 0.36 ^a^
	4	15.63 ± 0.36 ^d^	25.18 ± 0.39 ^a^	18.07 ± 0.43 ^c^	22.32 ± 0.43 ^b^	22.34 ± 0.44 ^b^	23.99 ± 0.77 ^a^
	7	20.56 ± 0.55 ^d^	28.19 ± 0.86 ^ab^	22.29 ± 0.43 ^c^	27.53 ± 0.42 ^b^	28.38 ± 0.37 ^ab^	29.50 ± 0.76 ^a^
Springiness	1	0.97 ± 0.02 ^a^	0.98 ± 0.02 ^a^	0.96 ± 0.02 ^a^	0.95 ± 0.02 ^a^	0.97 ± 0.02 ^a^	0.95 ± 0.02 ^a^
	4	0.87 ± 0.02 ^a^	0.86 ± 0.02 ^a^	0.89 ± 0.02 ^a^	0.87 ± 0.03 ^a^	0.86 ± 0.02 ^a^	0.85 ± 0.03 ^a^
	7	0.85 ± 0.02 ^a^	0.82 ± 0.02 ^a^	0.87 ± 0.02 ^a^	0.85 ± 0.02 ^a^	0.83 ± 0.02 ^a^	0.83 ± 0.02 ^a^
Cohesiveness	1	0.69 ± 0.03 ^a^	0.70 ± 0.02 ^a^	0.67 ± 0.02 ^a^	0.65 ± 0.03 ^a^	0.67 ± 0.03 ^a^	0.64 ± 0.03 ^a^
	4	0.56 ± 0.03 ^a^	0.45 ± 0.02 ^b^	0.44 ± 0.02 ^b^	0.48 ± 0.02 ^b^	0.44 ± 0.02 ^b^	0.43 ± 0.01 ^b^
	7	0.47 ± 0.03 ^a^	0.34 ± 0.03 ^b^	0.34 ± 0.01 ^b^	0.35 ± 0.03 ^b^	0.32 ± 0.02 ^b^	0.33 ± 0.01 ^b^
Chewiness	1	4.69 ± 0.19 ^b^	5.69 ± 0.28 ^b^	5.10 ± 0.02 ^b^	5.02 ± 0.17 ^b^	7.63 ± 0.60 ^a^	6.98 ± 0.69 ^a^
	4	7.59 ± 0.76 ^bc^	9.75 ± 0.51 ^a^	7.07 ± 0.33 ^c^	9.32 ± 0.53 ^a^	8.52 ± 0.50 ^ab^	8.63 ± 0.22 ^ab^
	7	8.23 ± 0.94 ^a^	7.86 ± 0.74 ^a^	6.60 ± 0.40 ^a^	8.18 ± 0.39 ^a^	7.63 ± 0.64 ^a^	7.76 ± 0.47 ^a^
Resilience	1	0.36 ± 0.02 ^a^	0.36 ± 0.03 ^a^	0.36 ± 0.02 ^a^	0.33 ± 0.02 ^a^	0.35 ± 0.02 ^a^	0.31 ± 0.02 ^a^
	4	0.23 ± 0.02 ^a^	0.19 ± 0.02 ^a^	0.20 ± 0.03 ^a^	0.20 ± 0.02 ^a^	0.18 ± 0.03 ^a^	0.17 ± 0.02 ^a^
	7	0.20 ± 0.02 ^a^	0.17 ± 0.03 ^a^	0.18 ± 0.01 ^a^	0.18 ± 0.02 ^a^	0.16 ± 0.02 ^a^	0.15 ± 0.02 ^a^

Data are presented as means ± SD of replicates. Distinct lowercase letters in the same column denote statistical significance (*p* < 0.05).

**Table 5:** Sensory attributes of cakes improved with TRP.

	Appearance	Flavor	Smell/Odor	Texture	Sweetness	Overall Quality
C1	4.59 ± 0.48 ^a^	4.36 ± 0.75 ^a^	4.13 ± 0.59 ^a^	4.40 ± 0.69 ^a^	4.31 ± 0.74 ^a^	4.42 ± 0.67 ^a^
C2	4.36 ± 0.77 ^a^	4.03 ± 1.00 ^a^	4.20 ± 0.73 ^a^	3.75 ± 1.02 ^a^	4.13 ± 0.92 ^a^	4.18 ± 0.83 ^a^
TRP3	4.08 ± 0.86 ^a^	4.31 ± 0.75 ^a^	4.08 ± 0.76 ^a^	4.15 ± 0.80 ^a^	4.15 ± 0.90 ^a^	4.15 ± 0.90 ^a^
TRP6	4.31 ± 0.75 ^a^	4.23 ± 0.73 ^a^	4.23 ± 0.73 ^a^	4.23 ± 0.60 ^a^	4.39 ± 0.65 ^a^	4.39 ± 0.65 ^a^
TRP9	4.54 ± 0.52 ^a^	4.15 ± 0.90 ^a^	4.39 ± 0.65 ^a^	4.00 ± 0.82 ^a^	4.15 ± 0.90 ^a^	4.39 ± 0.65 ^a^
TRP12	4.54 ± 0.66 ^a^	4.15 ± 0.80 ^a^	4.23 ± 0.73 ^a^	3.92 ± 0.76 ^a^	4.00 ± 0.91 ^a^	4.08 ± 0.76 ^a^

Data are presented as means ± SD of replicates. Distinct lowercase letters in the same column denote statistical significance (*p* < 0.05).

## Data Availability

The original contributions presented in the study are included in the article/Supplementary Materials; further inquiries can be directed to the corresponding author.

## Supplementary Materials

The following supporting information can be downloaded at: https://www.mdpi.com/article/10.3390/foods14061049/s1, Figure S1: A 10 cm hedonic unstructured scale; Table S1: The formulations of control and enriched cakes with terebinth seed proteins; Table S2: Descriptors, scale anchors, and descriptor definitions used in the quantitative descriptive analysis (QDA) of cake samples.
