# Supplementary material for: Development of Gluten-Free Cakes Using Protein Concentrate Obtained from Cold-Pressed Terebinth (Pistacia terebinthus L.) Oil By-Products
Source: Foods. 2025 Mar 19;14(6):1049. doi: 10.3390/foods14061049 (PMC11942047; doi:10.3390/foods14061049)
Supplement: Supplementary file 1 [file foods-14-01049-s001.zip › foods-3499290-supplementary.pdf]

Supplementary Information

Development of Gluten-Free Cakes Using Protein Concentrate Obtained from Cold-Pressed Terebinth (*Pistacia terebinthus* L.) Oil By-Products

Muhammed Ozgolet [1,*], Salih Karasu [1] and Muhammed Zahid Kasapoglu [2]

[1] Department of Food Engineering, Faculty of Chemical and Metallurgical Engineering, Yildiz Technical University, Davutpasa Campus, Esenler 34210, Istanbul, Turkey.

Corresponding author:

Muhammed Ozgolet

Department of Food Engineering, Faculty of Chemical and Metallurgical Engineering, Yildiz Technical University, Davutpasa Campus, Esenler 34210, Istanbul, Turkey.

Email: mozgolet@yildiz.edu.tr

Tel.: +90-531-380-0262

Table S1. The formulations of control, and enriched muffins with terebinth seed proteins

Ingredients	C1	C2	TRP3	TRP6	TRP9	TRP12
Rice flour (g)	0	50	48.5	47	45.5	44
Corn starch (g)	0	50	48.5	47	45.5	44
Sugar (g)	60	60	60	60	60	60
Egg (g)	50	50	50	50	50	50
Milk (g)	50	50	50	50	50	50
Sunflower oil (g)	50	50	50	50	50	50
Baking powder (g)	3.3	3.3	3.3	3.3	3.3	3.3
Xantham gum (g)	0	0.1	0.1	0.1	0.1	0.1
Protein (g)	0	0	3	6	9	12
Vanilin (g)	1.5	1.5	1.5	1.5	1.5	1.5
Wheat flour (g)	100	0	0	0	0	0

Table S2. Descriptors, scale anchors and descriptor definitions used in the quantitative descriptive analysis (QDA) of cake samples

Descriptor	Scale	Descriptor definition
**Appearance**	Mark their responses freely anywhere along the scale	• Pale or light yellow or uneven color distribution.<br>• Homogeneity as number and size of gas bubbles.<br>• Uniform rise and a smooth, slightly domed top.<br>• Presence of cracks.
**Texture**	Mark their responses freely anywhere along the scale	• Force applied to compress muffin via finger<br>• Speed of crumb recovery when removing pressure from a finger<br>• Difficulty when chewing the muffin completely

		• Sensed moisture in the mouth from muffin
**Flavor**	Mark their responses freely anywhere along the scale	• Bitterness<br>• Rancidity<br>• Any off-flavors<br>• Overpowering sweetness
**Odor/Smell**	Mark their responses freely anywhere along the scale	• Typical odor of baked food, consisting of fresh and sweet.<br>• The intensity of product-aromatic odor<br>• Off-odors like staleness, sourness, or burnt smells.
**Sweetness**	Mark their responses freely anywhere along the scale	• Perceived sugar intensity<br>• Bland or unappealing taste
**Overall quality**	Mark their responses freely anywhere along the scale	• Consumer acceptability<br>• Visually appealing, flavorful, and texturally pleasant<br>• Unappealing appearance, dryness, off-flavor

Sensory attributes of cakes in the quantitative descriptive analysis (QDA). The scale is unstructured. The scores were obtained through measurement using a ruler. The resultant scores being transformed to a 5-point scale.

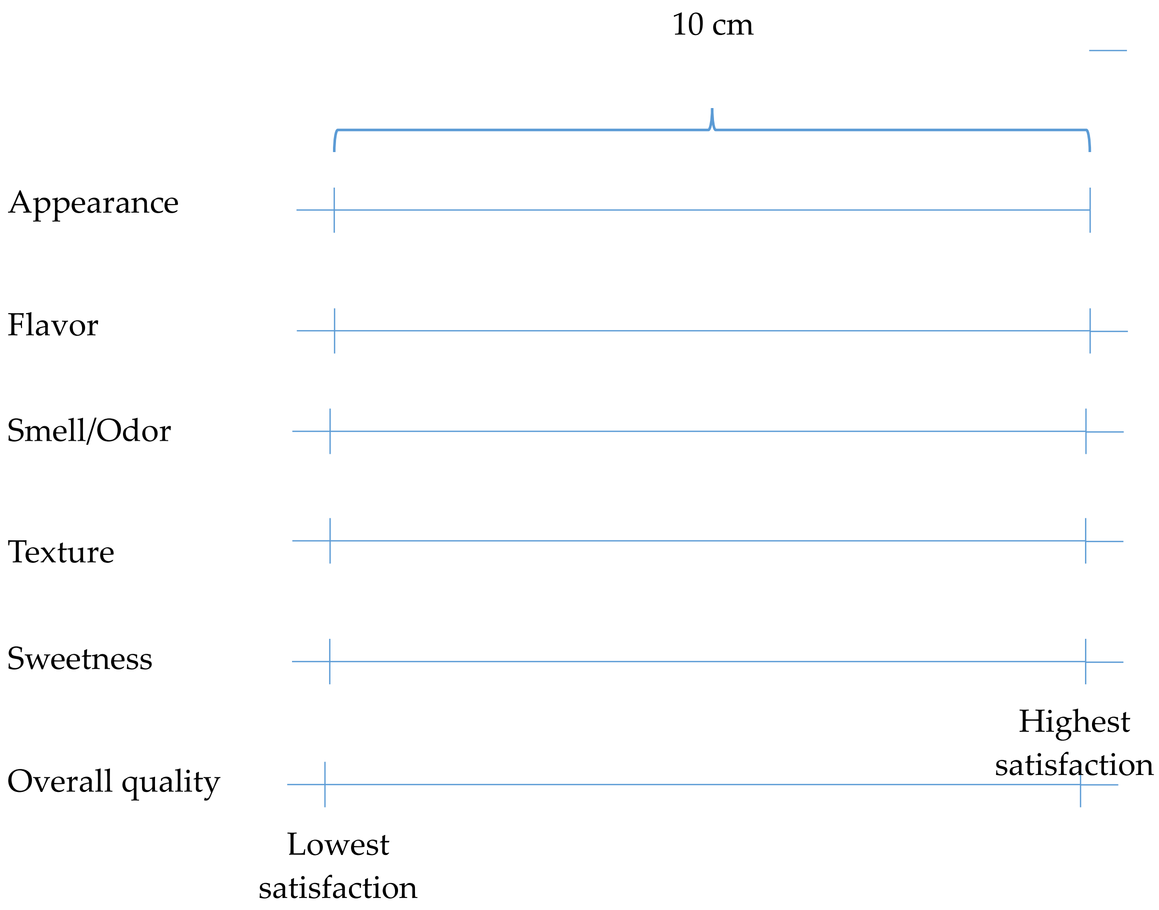

Figure S1. A 10-cm hedonic unstructured scale
